# Pituitary Extract

**Published:** 1913-12

**Authors:** 


PITUITARY EXTRACT. To sum up we may state that:

1. Pituitary extracts have a powerful effect in inducing and in strengthening uterine contractions.

2. The type of contractions induced is similar to that which occurs normally, although at first there may be a tendency to prolongation of the pains.

3. Such prolonged contractions result in slowing of the fœtal heart, but the child is seldom in danger.

4. When given in the late part of the first and in the second stage of full time labor the polarity of the uterine contractions is not interfered with, but in early abortions and early in the first stage a simultaneous spasm of the os may occur.

5. Its chief field of usefulness is in the first and second stages of labor, when there is delay due to feebleness of the pains alone or when combined with other complications, such as malpositions of head, malpresentations, multiple pregnancy, slight narrowing of the pelvis, etc.

6. In the induction of abortion, in the treatment of abortion already in progress, and in incomplete abortion, its action is so uncertain that it is not to be recommended, except in cases where the os is widely dilated.

7. In the induction of premature labor its effects are uncertain, but if sufficient dosage be given they may be good.

8. In the induction of labor at full term and after better results are obtained than in premature cases.

9. It gives good results in many cases of post-partum hæmorrhage, but is not superior to the various preparations of ergot. It has the power of sensitising the uterus, so as to allow these preparations to act more powerfully, the combination being most effective.

10. It is a useful adjunct in the treatment of placenta prævia, used in conjunction with rupture of the membranes, the use of hydrostatic dilators, or turning.—B. P. Watson, *Can. Med. Asso. Jour.*

